# Validation of the Comprehensive Feeding Practices Questionnaire among Brazilian Families of School-Aged Children

**DOI:** 10.3389/fnut.2015.00035

**Published:** 2015-11-03

**Authors:** Laís Amaral Mais, Sarah Warkentin, Maria do Rosário Dias de Oliveira Latorre, Susan Carnell, José Augusto de Aguiar Carrazedo Taddei

**Affiliations:** ^1^Department of Pediatrics, Discipline of Nutrology, Federal University of São Paulo (UNIFESP), São Paulo, Brazil; ^2^Department of Epidemiology, School of Public Health, University of São Paulo (USP), São Paulo, Brazil; ^3^Department of Psychiatry and Behavioral Sciences, Division of Child and Adolescent Psychiatry, Johns Hopkins University School of Medicine, Baltimore, MD, USA

**Keywords:** child nutrition, feeding behavior, child, validation studies, parent–child relations

## Abstract

**Introduction:**

Children’s eating behaviors are influenced by parents, who are the first nutritional educators. The comprehensive feeding practices questionnaire (CFPQ) was developed to measure feeding practices among parents, but has not yet been validated in Brazil, where child obesity rates are steeply increasing. The aim of the study was to test the validity of the CFPQ among Brazilian parents of school-aged children and propose a new version of the instrument.

**Methods:**

Transcultural adaptation included translation into Portuguese, back translation, content validity, testing for semantic equivalence, and piloting. Questionnaire data were obtained for 659 parents of 5- to 9-year olds. Confirmatory and exploratory factor analyses and psychometric analyses (tests for internal consistency, factor correlations, item-discriminant and convergent validity, and test–retest reliability) were conducted.

**Results:**

Confirmatory factor analysis demonstrated a poor fit of the data to the original 12-factor model. Exploratory factor analysis generated a 6-factor model composed of 42 items: healthy eating guidance, monitoring, restriction for weight control, restriction for health, emotion regulation/food as reward, and pressure. This factor solution was supported by internal consistency tests (α = 0.71–0.91) and factor correlations (ρ = −0.16 to 0.32). Item-discriminant and convergent validity tests showed that parents who used coercive practices had more overweight children and were more concerned about their child’s weight (ρ = 0.09–0.40). Test–retest reliability was acceptable (intraclass correlation coefficient = 0.45–0.77).

**Conclusion:**

Since parental practices are highly culturally and age group sensitive, it is essential to conduct careful evaluations of questionnaires when introduced into specific age groups within new cultural settings. This modified six-factor model of the CFPQ is valid to measure parental feeding behaviors of school-aged children in urban Brazilian settings.

## Introduction

The Brazilian population, like most societies in the world, is experiencing a nutritional transition, characterized by high intake of ultra-processed foods, such as artificial juice, soft drinks, and sugary snacks, over natural food intake, such as rice, beans, fruits, and vegetables (1, 2). This transition has increased body weight not just in adults but also in school-aged children, with the last national survey from 2008 to 2009 estimating that more than half of the Brazilian school-aged children from the urban Southeast region were overweight or obese (56.75%). These rates are comparable to those observed in developed societies, such as in the United States of America (USA) (19% obese) and Italy (62.0% obese or overweight) in 2009–2010 (3–5).

Overall, children learn very early about the food context and are highly influenced by the family. Parents are the child’s first nutritional educators, and shape children’s food environments, and thereby their eating behavior (e.g., food preferences, food intake self-regulation) via factors such as accessibility and availability of healthy and unhealthy food; modeling and teaching about nutrition; coercive practices, such as excessive control, restriction, pressure to eat; non-nutritive feeding practices such as using food as a reward; and responsiveness to the child’s internal signs of hunger and satiety (6–8). The determinants of parental feeding practices are multifactorial, including values, concerns, and responsibilities (9). In order to address obesity in Brazilian children, as well as to minimize the risk of nutritional inadequacy of food intake, it is, therefore, necessary to develop appropriate tools in order to study and understand more about parent feeding behavior (10).

Since 2001, the child feeding questionnaire (CFQ) has been the most widely used instrument to measure feeding practices, assessing parental restriction, monitoring, and pressure to eat (6, 11). In 2007, in order to develop a psychometrically valid scale assessing a more complete range of behaviors related to feeding practices, the comprehensive feeding practices questionnaire (CFPQ) was developed by US researchers. The CFPQ is a self-report instrument composed by 49 items distributed over 12 factors, with responses to be given on a 5-point Likert scale by parents of 2- to 8-year-old children (12, 13).

Despite the importance of understanding feeding practices in Brazil, a young, culturally diverse country where feeding practices may be different than in other countries due to factors, such as tradition, religion practices, and social demands (14, 15), there is a lack of validated instruments in Portuguese to quantify parental feeding behaviors and styles. Validation of instruments for different populations is essential because of cultural variation, which could impact parental feeding practices and, consequently, children’s eating behavior and weight status (12, 16, 17). Further, adapted instruments are better understood by the target population, ensuring the accuracy and quality of the information (18).

The aim of the current study was, therefore, to test the validity of the CFPQ within a large sample of Brazilian parents of 5- to 9-year olds enrolled in private schools, and to derive an optimized version of the instrument. This age group is especially interesting because as the child matures and starts to eat outside the home environment, relationships with parental feeding change. Although these children are more independent than younger ages and more exposed to external influences, such as school, friends, advertisement, and other environment determinants, they are still very affected by parents’ attitudes and practices regarding eating behavior and food choice (19–21).

## Materials and Methods

### Overview

This study of Brazilian parents of 5- to 9-year olds was composed of two phases: (1) transcultural adaptation of the CFPQ and (2) psychometric analyses including confirmatory and exploratory factor analyses, and tests for internal consistency, factor correlations, item-discriminant and convergent validity, and test–retest reliability.

To estimate sample size, we used the Gorsuch (22) criteria for acceptable factor analysis, which suggests inclusion of at least five participants per question, or a minimum of 200 respondents (22). Since the CFPQ is composed of 49 items, this estimation resulted in 245 individuals. Accounting for 10% dropout, we, therefore, aimed to recruit 270 participants, in total.

For practical reasons, participants were recruited from private schools in the cities of Campinas and São Paulo, via email or telephone, followed by a meeting with the schools’ headmaster and/or coordinator. Seventeen of the 48 contacted schools accepted the invitation to participate in the study. Two of these schools participated in a pilot study, and the remaining 15 participated in the main study. One of these 15 remaining schools also participated in a test–retest reliability procedure.

This research received ethical approval from the Federal University of São Paulo (UNIFESP) Ethics Committee.

### Phase 1: Transcultural Adaptation of CFPQ

Study researchers made contact with the corresponding author of the original scale asking for permission to translate and validate it into Portuguese, and agreement was obtained. Transcultural adaptation was initialized with the translation of the CFPQ into Portuguese by three pediatric nutrition researchers fluent in English. A back translation was then made by a translator blind to the original version of the CFPQ. The same three researchers then translated the questionnaire into Portuguese a second time, in order to improve understanding and to reduce confusion regarding terminology (23).

After this step, the Portuguese version of the CFPQ was emailed to 11 dietitians, to evaluate its content validity. All the comments/suggestions were compiled and discussed in a 2-h expert panel session, resulting in a slightly modified version of the questionnaire (e.g., changes in the order of some sentences, and replacement of specific words, such as “to regulate” for “to control” and “to discuss” for “to talk”). Semantic equivalence of the new version was then tested in 11 parents of index children drawn at random from two classrooms within one of the selected schools, and some items were modified based on parents’ answers/understanding (e.g., replacement of specific words, such as “to ensure” for “to confirm”).

### Phase 2: Validation of CFPQ

First, in order to expose any difficulties with questionnaire completion and increase data accuracy, we conducted a pilot study in two of the participating schools. This identified several aspects that needed to be changed to increase comprehension and specificity (e.g., changes in the order of some sentences’ and replacement of specific words/expressions, such as “to encourage” for “to promote” and “the food tastes good” for “the food is tasty”).

After piloting, we conducted the main study. Survey packets including information letters, consent forms, and self-administered questionnaires were left in each classroom at each participating school to be distributed to eligible children, with instructions to bring them home to be completed by one of the parents within 2 weeks. In one of the schools, the survey packets were administered and completed by parents before a parents and teachers meeting. Parent-report anthropometric information was obtained within the survey packet. All returned questionnaires were examined for inconsistencies and missing answers using a consistent protocol performed by two trained researchers. Parents were called up to three times to resolve ambiguous responses. In case of missing phone numbers or parents not picking up, the data were entered as “missing” in the database. Missing data in the CFPQ led to child exclusion.

Finally, one of the participant schools was selected to examine test–retest reliability. After 2 weeks, respondent parents received the CFPQ to be answered again. This interval was chosen to limit the likelihood that feeding practices would have changed with child age, and to reduce the chance of participants’ responding primarily based on recall of their first set of answers (24). From a total of 97 distributed questionnaires, we received 78 completed pairs (80.4%).

### Statistical Analysis

Confirmatory, followed by exploratory factor analysis, was conducted on the 12-factor original model (12) using oblique rotation (Promax) since factors were hypothesized to correlate. Items were treated as ordinal and, to avoid over- or under-extraction of factors, we used the Kaiser criteria (the eigenvalues-greater-than-one rule) (25), and required coefficients >0.3 in the correlation matrix (26). Scree plots were additionally examined. Internal consistency of items within each identified factor was tested using Cronbach’s alpha, with values higher than 0.70 considered acceptable. To check for overlap between factors, we ran Spearman’s correlations, with values ≥0.85 considered indicative of strong overlap (26).

Item-discriminant validity was assessed by running Mann–Whitney’s tests, comparing scale means with indices of children’s food intake. For this, we used low and high intakes of ultra-­processed food (i.e., fast food, instant noodles, soft drink, artificial juice, chips, sugared snacks, breakfast cereal, chocolate milk, crackers/biscuits/cakes with and without topping, ice cream/popsicles, dairy desserts, and processed meat) as determined by median intake from the food frequency questionnaire (FFQ).

Convergent validity was assessed by running Spearman’s correlations between both original and proposed scales and three related attitude scales derived from Birch et al. (6). Concern about child’s overweight (three items) and perceived responsibility for feeding (three items) were identical to the “concern about child weight” and the “perceived responsibility” scales of the CFQ, respectively. Concern about child’s underweight (three items) was adapted from the CFQ by changing the words “overweight” to “underweight” and “diet” to “eat more” (6, 12).

Finally, test–retest reliability was assessed by calculating intraclass correlation coefficients (ICCs), for each factor within both the original and the proposed factor solution, with scales considered reliable if ICC values were >0.40. Also, Bland–Altman’s graphs were created using MedCalc for Windows, version 15.2.2 (27).

Data were entered twice and analyzed using Stata version 12.0 (28), with the help of two trained assistant researchers.

## Results

### Participants

**Figure 1:**
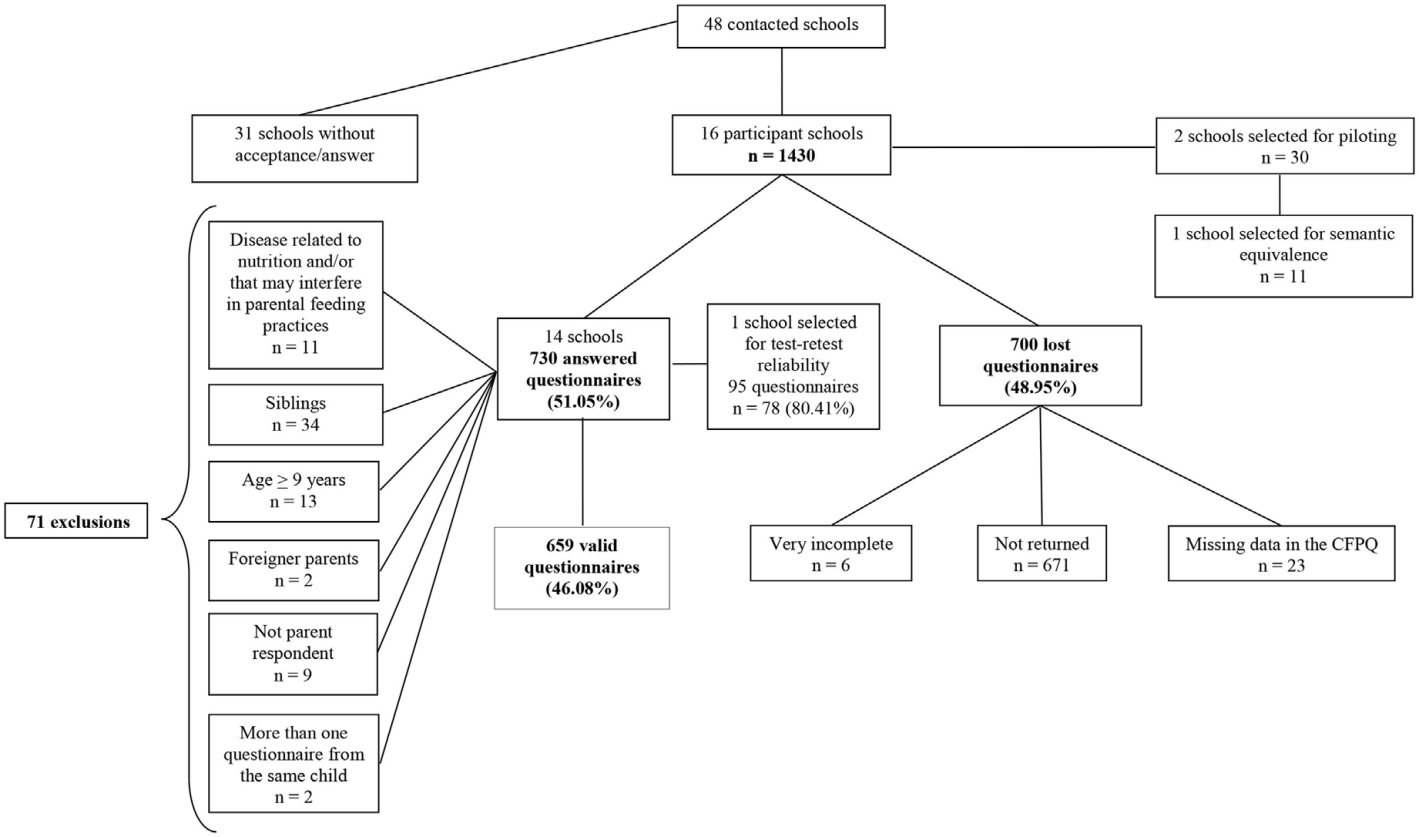
**Flowchart of losses and exclusions**.

Of the total of 1430 survey packets distributed, we received 730 completed questionnaires (51.0%). Of the remaining 700, 671 were not returned, 23 had missing data on the CFPQ, and six had essential demographic and anthropometric information missing. Of the completed 730 questionnaires, 34 were excluded due to index children having siblings in the same age group, to avoid sample over-representation of those family units (in case of siblings, the youngest child was included; in case of twins, the child whose name began with the earliest letter in the alphabet was included), 13 for not being within the eligible age group, and 11 for reporting diseases related to nutrition and/or other conditions that might interfere with parental feeding practices, such as lactose intolerance or cow’s milk protein allergy (*n* = 4), celiac disease (*n* = 2), diabetes mellitus (*n* = 1), hepatic insufficiency (*n* = 1), visual deficiency (*n* = 1), Landau–Kleffner syndrome (*n* = 1), and autism (*n* = 1). We also excluded cases where questionnaires were completed by individuals other than parents (*n* = 9), where parents had a mother language other than Portuguese (*n* = 2), or where parents answered more than one questionnaire for the same child (*n* = 2). Following exclusions, there were 659 valid questionnaires (effective 46.1% response rate) (Figure 1).

Table 1 describes demographic and anthropometric characteristics of the final sample, which was 53.7% girls with a mean age of 6.35 (±1.05 SD) years and 35.9% overweight. Around 90% of the respondents were mothers with an average age of 38.9 years. The majority had graduated from college (86.2%) and over a third (34.9%) were overweight or obese. Most of the families (56.1%) earned more than 16 times the Brazilian minimum wage, corresponding to a monthly income of US$5148.32.

**Table 1:** **Prevalence rates of the demographic and anthropometric characteristics of school-aged children enrolled at private schools of São Paulo and Campinas, Brazil, 2014**.

Demographic and anthropometric characteristics	Category	*n* (%)
Sex	Male	305 (46.28)
	Female	354 (53.72)
BMI/age *z*-score	Extremely underweight	10 (1.58)
	Underweight	11 (1.74)
	Normal weight	385 (60.82)
	Overweight	149 (23.54)
	Obese	65 (10.27)
	Extremely obese	13 (2.05)
Respondent	Mother	596 (90.44)
	Father	63 (9.56)
Maternal education	College completed	567 (86.17)
	College incomplete	44 (6.69)
	High school completed	38 (5.78)
	High school incomplete	5 (0.76)
	Middle school completed	2 (0.30)
	Middle school incomplete	2 (0.30)
Family’s income	Until 5 minimum wage	43 (6.91)
	From 6 to 10 minimum wage	113 (18.17)
	From 11 to 15 minimum wage	117 (18.81)
	From 16 to 20 minimum wage	117 (18.81)
	More than 20 minimum wage	232 (37.30)
Maternal BMI	Underweight	12 (1.84)
	Normal weight	424 (65.13)
	Overweight	172 (26.42)
	Obese	43 (6.61)

*BMI, body mass index*.

*Brazilian minimum wage in 2014: R$724.00 (US$321.77)*.

### Factor Analysis

**Figure 2:**
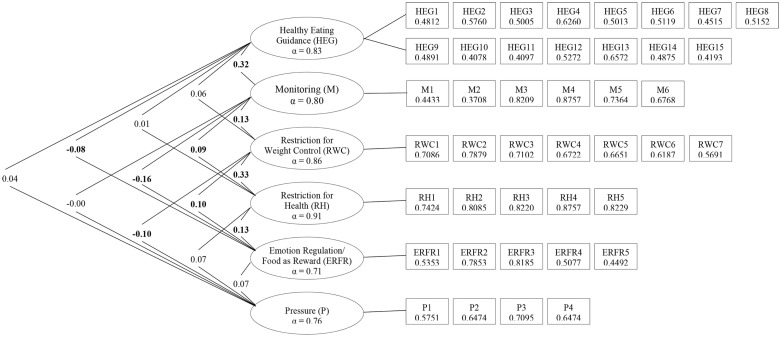
**Spearman’s correlations between factors, Cronbach’s alpha for each sub-scale, and factor loadings for each item**. *Note*: values on the left side of the table are correlations (Spearman’s rho), with significant correlations (*p* < 0.05) in bold. Values in ovals are Cronbach’s alpha (α) for each derived sub-scale (all over 0.70). Values in boxes give factor loadings from exploratory factor analysis (all over 0.30).

Since the initial confirmatory factor analysis did not confirm the factor structure of the original CFPQ’s model in our sample, we conducted an exploratory factor analysis. Both the matrix model and scree plot (data not shown) indicated that six factors should be extracted. The exploratory factor analysis also demonstrated that seven items should be excluded: items 2, 3, 5, 18, and 49 due to factor loadings <0.3, and items 1 and 4 due to negative factor loadings. Amongst these items was the entire “child control” factor from the original model. The final 6-factor model comprised 42 items (Supplementary Material). Factor loadings for all items were higher than 0.3 (0.41–0.88) (Figure 2), and each factor explained a minimum of 10% of the variance.

Derived factors were as follows:

- *Healthy eating guidance* (15 items). This factor was composed of items within the original “encourage balance and variety,” “involvement,” “modeling,” “and teaching about nutrition” (minus one item) factors plus two items from the original “environment” factor. Assesses how parents guide their child through encouragement, modeling, and teaching about nutrition, as well as the influence of parents’ involvement and healthy environments.
- *Monitoring* (six items). This factor incorporated all items in the original “monitoring” factor as well as two items from the original “environment” factor. Assesses how much parents keep track of unhealthy food their child eats.
- *Restriction for weight control* (seven items). This factor replicated the entire original factor, except for one item. Assesses the degree to which parents restrict their child’s food intake to control their child’s weight status.
- *Restriction for health* (five items). This factor is composed of all items within the entire original “restriction for health” factor plus one item from the original “restriction for weight control” factor. Assesses how much parents restrict their child’s food intake to influence their child’s health.
- *Emotion regulation/food as reward* (five items). This factor was a combination of items within both original factors minus one item from “food as reward.” Assesses parents’ use of food to regulate child’s emotions and/or as reward for desirable behaviors.
- *Pressure* (four items) replicates the entire original pressure factor, and assesses the degree to which parents use pressure to make their child eat more and/or a specific food.

Spearman’s correlations revealed low correlations between factors (ρ = −0.16 to 0.32) indicating no overlap (26), and Cronbach’s alpha values were all higher than 0.70 (0.71–0.91) (Figure 2).

Item-discriminant validity between factors from the original and proposed scales, and child’s ultra-processed food intake, is represented in Table 2. For the original scale, “encourage balance and variety,” “environment,” “involvement,” “modeling,” “monitoring,” “child control,” “emotion regulation,” “food as reward,” and “restriction for health” factors were able to differentiate children exhibiting low and high ultra-processed food intake. For the proposed scale, “healthy eating guidance” and “monitoring” were significantly associated with lower intake of ultra-processed food, while “restriction for health” and “emotion regulation/food as reward” were significantly associated with higher ultra-processed food intake.

**Table 2:** **Item-discriminant validity on the CFPQ by ultra-processed food intake among school-aged children of private schools of São Paulo and Campinas, Brazil, 2014**.

Factors	Ultra-processed food		*p*^a^
		
	Low intake	High intake	
		
	M (SD)	M (SD)	
**Original scale**			
Child control	2.56 (0.63)	2.77 (0.69)	<**0.001**
Emotion regulation	1.22 (0.40)	1.30 (0.50)	**0.030**
Encourage balance and variety	4.76 (0.38)	4.67 (0.45)	**0.007**
Environment	4.34 (0.62)	3.95 (0.71)	<**0.001**
Food as reward	1.57 (0.81)	1.76 (0.95)	**0.009**
Involvement	3.98 (0.93)	3.72 (0.99)	<**0.001**
Modeling	4.67 (0.46)	4.57 (0.56)	**0.015**
Monitoring	4.49 (0.67)	4.31 (0.75)	<**0.001**
Pressure	3.31 (0.97)	3.30 (1.05)	0.745
Restriction for health	3.55 (1.27)	3.82 (1.20)	**0.005**
Restriction for weight control	2.42 (0.91)	2.50 (0.96)	0.348
Teaching about nutrition	4.48 (0.64)	4.47 (0.64)	0.783
**Proposed scale**			
Healthy eating guidance	4.52 (0.41)	4.38 (0.43)	<**0.001**
Monitoring	4.39 (0.62)	4.07 (0.70)	<**0.001**
Restriction for weight control	2.25 (0.96)	2.31 (1.01)	0.519
Restriction for health	3.57 (1.26)	3.82 (1.18)	**0.007**
Emotion regulation/food as reward	1.32 (0.44)	1.45 (0.54)	**0.002**
Pressure	3.31 (0.06)	3.30 (0.06)	0.745

*M, means; *p*, *p* value. Values in bold are significant (*p* < 0.05)*.

*^a^Mann–Whitney’s test*.

Table 3 shows correlations between all factors and scales measuring related attitudes. For the original scale, perceived responsibility was negatively correlated with all the factors related to positive practices, such as “encourage balance and variety,” “environment,” “involvement,” “modeling,” “monitoring,” and “teaching about nutrition,” and “child control.” Parental concern about child’s overweight was positively associated with “restriction for health” and “restriction for weight control,” while concern about underweight was positively associated with “pressure.” Perceived responsibility for feeding was positively correlated with scores on the proposed factors “healthy eating guidance,” “monitoring,” “restriction for health,” and “pressure.” Parental concern about their child’s weight status also showed significant associations, such that “restriction for health” and “pressure” were positively correlated with concern about underweight, and “monitoring,” “restriction for weight control,” and “restriction for health” were positively correlated with concern about overweight.

**Table 3:** **Convergent validity between the original and proposed scales and parents’ concern about over- and underweight and perceived responsibility for feeding**.

Factors	Perceived responsibility for feeding	Concern about overweight	Concern about underweight
	
	ρ (*p*)	ρ (*p*)	ρ (*p*)
**Original scale**			
Child control	−**0.24 (<0.001)**	−**0.10 (0.008)**	−**0.09 (0.022)**
Emotion regulation	−0.04 (0.282)	0.02 (0.595)	−0.01 (0.755)
Encourage balance and variety	**0.29 (<0.001)**	0.01 (0.726)	0.03 (0.500)
Environment	**0.15 (<0.001)**	0.07 (0.086)	−0.05 (0.224)
Food as reward	0.02 (0.567)	0.02 (0.642)	−0.03 (0.490)
Involvement	**0.13 (0.001)**	0.07 (0.086)	0.03 (0.460)
Modeling	**0.19 (<0.001)**	0.05 (0.234)	0.05 (0.161)
Monitoring	**0.28 (<0.001)**	**0.08 (0.043)**	−0.00 (0.932)
Pressure	**0.12 (0.002)**	−0.10 (0.017)	**0.21 (<0.001)**
Restriction for health	0.07 (0.096)	**0.25 (<0.001)**	**0.09 (0.026)**
Restriction for weight Control	0.05 (0.254)	**0.41 (<0.001)**	0.02 (0.681)
Teaching about nutrition	**0.12 (0.002)**	−0.00 (0.994)	−0.00 (0.989)
**Proposed scale**			
Healthy eating guidance	**0.25 (<0.001)**	0.06 (0.105)	0.05 (0.243)
Monitoring	**0.23 (<0.001)**	**0.11 (0.004)**	−0.03 (0.475)
Restriction for weight control	0.03 (0.448)	**0.40 (<0.001)**	−0.00 (0.980)
Restriction for health	**0.08 (0.043)**	**0.26 (<0.001)**	**0.09 (0.018)**
Emotion regulation/food as reward	0.00 (0.996)	−0.00 (0.971)	−0.03 (0.463)
Pressure	**0.12 (0.002)**	−0.09 (0.017)	**0.21 (<0.001)**

*ρ, correlation coefficient; *p*, *p* value. Values in bold are significant (*p* < 0.05)*.

*Spearman’s test*.

Test–retest reliability analyses demonstrated ICC values ranging from 0.27 to 0.78 for the original scale and ICCs from 0.45 to 0.77 for the proposed scale. Satisfactory reliability was also verified by Bland–Altman’s graphs, which demonstrated randomness (data not shown).

## Discussion

The present paper presents the adaptation and validation of a Portuguese version of the CFPQ in a large sample of urban Brazilian parents of school-aged children. Transcultural adaptation of the questionnaire led to some small changes in sentence order and in some verbs and food names. Exploratory factor analysis produced a 6-factor model of parental feeding practices (“healthy eating guidance,” “monitoring,” “restriction for weight control,” “restriction for health,” “emotion regulation/food as reward,” and “pressure”) with a better fit for our sample.

Most of the items loaded as expected. For example, “pressure” had exactly the same composition as the original factor. This parental practice is related to lower weight in children and higher parental concern about child weight (8, 29). It has also been associated with poorer intake regulation (6), potentially resulting from reduced desirability of the food the child is being pressured to eat (30).

The original factors “emotion regulation” and “food as reward,” with the exception of one excluded item, loaded together in our factor solution, suggesting that these practices tend to cluster together in our population of interest. The use of food to regulate a child’s emotional state may lead the child to learn to use food in order to alleviate or distract from their emotions (31, 32), and there is evidence that using food as reward may make the “reward” food more desirable and the food intended for consumption (usually vegetables) less desirable (33).

The most widely studied feeding practice, restriction, maintained its original structure such that separate factors emerged for “restriction for weight control” and “restriction for health.” The only exception was item 39, which loaded on the former factor in the original model but on the latter factor in the current analysis. The distinction between these two factors may be important, because, for example, restriction for health may be associated with teaching the child healthy eating habits for the future, while restriction for weight control could potentially engender weight concern and disordered food-related attitudes among children (14, 34). Certainly there is some evidence that authoritarian styles of restriction to control unhealthy food intake in children may make restricted food becomes more desirable leading to over-consumption in permissive environments, resulting in excessive weight gain in the long term (35, 36).

“Monitoring” is a practice that could be interpreted as negative, if associated with rigid, authoritarian parental control, or positive, if seen as a more authoritative, flexible way for parents to limit their children’s intake, since young children do not have full autonomy to make wise decisions (11, 14, 30). Our results support the latter interpretation, since items in the original “monitoring” factor loaded with two additional items from the original factor “environment,” which is a beneficial parent feeding strategy. It is worth noting that both items from this factor that loaded onto our new factor “monitoring” reflected the availability of unhealthy food in the house (reverse scored), while the other two items, concerning the availability of healthy food at home, both loaded on the new factor “healthy eating guidance.”

The final extracted factor was “healthy eating guidance” – a combination of the entire original factors “encourage balance and variety,” “involvement,” “modeling,” and “teaching about nutrition” (except one excluded item) and half of the “environment” factor, all of which measure child-centered, positive feeding practices. These kinds of practices, i.e., reasoning, encouraging, complimenting, being a good example, and providing healthy food, allow the child to develop good internal self-regulation of intake (30, 37). The joint loading that we observed in our sample suggests that these practices do not occur in isolation in this population (11), but instead form a constellation of behaviors reflecting an overall pattern of positive parent feeding practices. Although negative practices have been paid the most attention in the literature, it is also important to focus on feeding practices associated with healthy eating habits and weight status in ­children (12, 33).

Seven items loaded below 0.3 in the matrix model, including the entire “child control” (items 1–5) and so, were excluded. A probable explanation for this is that school-aged children become more autonomous through the years, which leads parents to gradually transfer control to them (38–40). Thus, the parental feeding practice “child control” is less applicable in this age group. Low factor loadings might also be explained by differential parent–child relationships in Brazilian food culture (12, 16, 17), since this factor remained in all other validation studies of the CFPQ developed in other cultures and age groups (11, 14, 17, 41).

Item 18, which assesses the withholding of sweets/dessert from the child in response to bad behavior, was also excluded due to low factor loading, suggesting that this practice does not tend to be paired with offering their child’s favorite food to reward good behavior. Similar to the Malay validation of the CFPQ, item 49 (“I tell my child what to eat and what not to eat without explanation”) also did not load above 0.3 on any factor, including the factor it loaded on in the original CFPQ, “teaching about nutrition.” The low loading we observed may be because this item reflects a rigid form of parental control paired with a lack of concern about involving the child in the feeding interaction, which seems qualitatively different from the other two items from this scale (17).

Internal consistency testing demonstrated high reliability, with each factor, especially those with few modifications from the original and those with more items, demonstrating high Cronbach’s alpha values. The lowest (but still adequate value) was for “emotion regulation/food as reward” (0.71), which is a combination of two of the original factors each containing five items. In contrast to our findings, Cronbach’s alpha values for some of the original factors and for the factors emerging from other validation studies were lower than desirable (0.70), which reinforces the adequacy of our results for our particular population of interest (12, 14, 17).

Although parental feeding practices are hypothesized to correlate, correlations between factors were not substantial, indicating that each factor captures specific practices. The highest correlation was found between “restriction for health” and “restriction for weight control.” Both of these factors represent conceptually close practices, since having good health is associated with having a healthy body weight and a correlation was also observed by Musher-Eizenman and Holub (*r* = 0.34). Notably, parents do not spontaneously distinguish the motivation for restriction when they use or report this practice (12, 14).

“Healthy eating guidance” and “monitoring” were also moderately correlated (0.32). This was likely because both factors are characterized by child-centered and positive feeding practices, and parents who use positive practices tend to use them not in isolation (11, 42). In general, items regarding positive practices showed positive correlations, with the same pattern for negative practices. On the other hand, as also observed by Musher-Eizenman and Holub, negative correlations emerged between positive and negative practices, e.g., “healthy eating guidance” and “monitoring” with “emotion regulation/food as reward” (−0.08 and −0.16, respectively) (12).

Unlike some of the previous CFPQ validation studies (11, 12, 14), we conducted test–retest reliability analyses and found support for reproducibility of the proposed factors, with ICC values >0.4 for all factors. Notably, this was not so for the original factors in our sample, which gave inadequate ICC values for “emotion regulation” and “monitoring.”

We also tested item-discriminant validity, which evaluates an instrument’s discrimination capability between well-known different groups, using a construct indicator, in this case children’s ultra-processed food intake. As expected, the use of “restriction for health” and “emotion regulation/food as reward” was both related to higher intake of ultra-processed food. Both of these practices may decrease responsivity to internal satiety cues, thereby increasing energy intake, especially from sweet and fatty foods, and potentially leading to excessive weight gain (31, 33, 36). In contrast, the positive parental practices represented by both “healthy eating guidance” and “monitoring,” were positively associated with lower intake of ultra-processed food, likely reflecting healthier home food environments and responsive feeding styles among these families (11, 12, 30). Although “restriction for weight control” and “pressure” were not significantly associated with the selected indicator, it was notable that this was also so for the original factors. Interestingly, the original factor “teaching about nutrition” also did not discriminate between children with high and low ultra-processed food intake, suggesting that didactic attempts to promote healthy eating may be less effective than other means (12, 30).

Finally, convergent validity analyses demonstrated, as expected, that higher perceived responsibility for feeding was associated with the child-centered and positive parental practices captured by the “healthy eating guidance” and “monitoring” factors. Further, parents who reported more concern about their child being overweight also reported more restrictive feeding practices, whereas those who were concerned about their child being underweight reported more pressure to eat. There were no significant associations, however, between “emotion regulation” and “food as reward” with parental perceived responsibility and concerns. Notably, the same pattern of correlations was largely found for the original scales except that “child control” showed negative correlations with parental responsibility and concern (9, 11, 12, 14, 39).

We had a response rate of 46.08%, which was adequate for our statistical approach and in the expected range of 38–48% for surveys of this nature (43). Notably, other validation studies have also reported similar response rates (40, 44). Although 90.44% of the respondents were mothers, this was not seen as a limitation, since they are considered “nutrition gate-keepers” due to being the parent most likely to choose, purchase, prepare, and serve food to the child. Hence, mothers are usually the parent accompanying the child during mealtimes, and are thereby largely responsible for feeding practices (13).

Our sample was composed of Brazilian families with relative high income and education. This could be seen as a limitation, since it reduces the generalizability of the results to the wider population, which, due to Brazil’s territorial extension and continental features, is largely low in socioeconomic status. However, a significant portion of the Brazilian population earns high incomes and the results of our study are comparable to this sector. Although obesity is more likely in low-income populations, we believe research in relatively high-income communities is also important, because these parents have the possibility of buying and offering whatever they wish to their children, whereas low-income parents have their purchasing power comparatively contained.

Other validations of the CFPQ have been conducted in the USA (English, 2–8 years old, *n* = 152) (12), France (French, 4–7 years old, *n* = 122) (44), Norway (Norwegian, 10–12 years old, *n* = 963) (14), Iran (Persian, 3–5 years old, *n* = 150) (41), Malaysia (Malay Language, 7–9 years old, *n* = 397) (17), and New Zealand (English, 4–8 years old, *n* = 1013) (11). However, our own study makes some important additions to the literature because, of these, only one focused specifically on school-aged children, and did not require translation since English was the mother language (11). None included Portuguese translation or tested item-discriminant and convergent validity, which are recommended for adequate instrument validation. Most of them were conducted in smaller samples.

Notably, all of the validation studies, including our own, resulted in a slightly modified version of the questionnaire, likely due to differences in cultural background and/or age group (16, 20, 21). In our particular analyses, the collapsing of factors, such as “healthy eating guidance” and “emotion regulation/food as reward” led to the loss of some subscales and, potentially, the ability to specifically detect which behaviors are most effectual (11). However, our solution also produced more robust factors, with higher internal validity, and all factors emerged from the data were defined by at least four items, as recommended (26). Moreover, the validation process resulted in the questionnaire being reduced from 49 to 42 items, which decreases response burden (11).

To conclude, the present validation study used multiple methods including transcultural adaptation, test–retest reliability, factor correlations, and internal, item-discriminant, and convergent validity, to derive a modified Portuguese version of the CFPQ into for use with middle- and high-income and well-educated parents of school-aged children. Since the proposed scale was demonstrated to be valid and reliable, we recommend its use to assess parental feeding practices in a Brazilian setting.

## Author Contributions

LM contributed for the selection of study design, participated in the data gathering and data entering, performed the data analysis and interpretation, wrote the article, and approved the final version to be published. SW contributed for the selection of the study design, participated in the data gathering and data entering, and contributed to the data analysis and interpretation, writing of the article, and approved the final version to be published. ML suggested and supervised the data analysis and interpretation, reviewed the article, and approved the final version to be published. SC contributed to the interpretation of the data, writing and revising the article critically, and approved the final version to be published. JT selected the study design, supervised the data gathering and data entering, contributed to the data analysis and interpretation, reviewed the article, and approved the final version to be published.

## Conflict of Interest Statement

The authors declare that the research was conducted in the absence of any commercial or financial relationships that could be construed as a potential conflict of interest.
