# Supplementary material for: Validation of the Comprehensive Feeding Practices Questionnaire among Brazilian Families of School-Aged Children
Source: Front Nutr. 2015 Nov 3;2:35. doi: 10.3389/fnut.2015.00035 (PMC4630658; doi:10.3389/fnut.2015.00035)
Supplement: Supplementary file 1 [file Data_Sheet_1.DOCX]

**Appendix 1.** Factors and items from the proposed scale and original factors.

| **Factors and Items from the Proposed Scale** | **Original Factors** |
| --- | --- |
| ***Healthy Eating Guidance*** |  |
| 9. Do you encourage this child to eat healthy foods before unhealthy ones?* | Encourage Balance and Variety |
| 10. I encourage my child to try new foods.** | Encourage Balance and Variety |
| 11. I tell my child that healthy food tastes good.** | Encourage Balance and Variety |
| 12. I encourage my child to eat a variety of foods.** | Encourage Balance and Variety |
| 13. Most of the food I keep in the house is healthy.** | Environment |
| 15. A variety of healthy foods are available to my child at each meal served at home.** | Environment |
| 20. I involve my child in planning family meals.** | Involvement |
| 21. I allow my child to help prepare family meals.** | Involvement |
| 22. I encourage my child to participate in grocery shopping.** | Involvement |
| 23. I model healthy eating for my child by eating healthy foods myself.** | Modeling |
| 24. I try to eat healthy foods in front of my child, even if they are not my favorite.** | Modeling |
| 25. I try to show enthusiasm about eating healthy foods.** | Modeling |
| 26. I show my child how much I enjoy eating healthy foods.** | Modeling |
| 47. I discuss with my child why it’s important to eat healthy foods.** | Teaching about Nutrition |
| 48. I discuss with my child the nutritional value of foods.** | Teaching about Nutrition |
| ***Monitoring*** |  |
| 14. I keep a lot of snack food (potato chips, Doritos, cheese puffs) in my house.****R** | Environment |
| 16. I keep a lot of sweets (candy, ice cream, cake, pies, pastries) in my house.****R** | Environment |
| 27. How much do you keep track of the sweets (candy, ice cream, cake, pies, pastries) that your child eats?* | Monitoring |
| 28. How much do you keep track of the snack food (potato chips, Doritos, cheese puffs) that your child eats? | Monitoring |
| 29. How much do you keep track of the high-fat foods that your child eats?* | Monitoring |
| 30. How much do you keep track of the sugary drinks (soda/pop, kool-aid) this child drinks?* | Monitoring |
| ***Restriction for Weight Control*** |  |
| 40. I encourage my child to eat less so he/she won’t get fat.** | Restriction for Weight Control |
| 41. I give my child small helpings at meals to control his/her weight.** | Restriction for Weight Control |
| 42. If my child eats more than usual at one meal, I try to restrict his/her eating at the next meal.** | Restriction for Weight Control |
| 43. I restrict the food my child eats that might make him/her fat.** | Restriction for Weight Control |
| 44. There are certain foods my child shouldn’t eat because they will make him/her fat.** | Restriction for Weight Control |
| 45. I don’t allow my child to eat between meals because I don’t want him/her to get fat.** | Restriction for Weight Control |
| 46. I often put my child on a diet to control his/her weight.** | Restriction for Weight Control |
| ***Restriction for Health*** |  |
| 35. If I did not guide or regulate my child’s eating, s/he would eat too much of his/her favorite foods.** | Restriction for Health |
| 36. If I did not guide or regulate my child’s eating, he/she would eat too many junk foods.** | Restriction for Health |
| 37. I have to be sure that my child does not eat too much of his/her favorite foods.** | Restriction for Health |
| 38. I have to be sure that my child does not eat too many sweets (candy, ice cream, cake, or pastries).** | Restriction for Health |
| 39. I have to be sure that my child does not eat too many high-fat foods.** | Restriction for Weight Control |
| ***Emotion Regulation/Food as Reward*** |  |
| 6. When this child gets fussy, is giving him/her something to eat or drink the first thing you do?* | Emotion Regulation |
| 7. Do you give this child something to eat or drink if s/he is bored even if you think s/he is not hungry?* | Emotion Regulation |
| 8. Do you give this child something to eat or drink if s/he is upset even if you think s/he is not hungry?* | Emotion Regulation |
| 17. I offer sweets (candy, ice cream, cake, pastries) to my child as a reward for good behavior.** | Food as Reward |
| 19. I offer my child his/her favorite foods in exchange for good behavior.** | Food as Reward |
| ***Pressure*** |  |
| 31. My child should always eat all of the food on his/her plate.** | Pressure |
| 32. If my child says, ‘‘I’m not hungry,’’ I try to get him/her to eat anyway.** | Pressure |
| 33. If my child eats only a small helping, I try to get him/her to eat more.** | Pressure |
| 34. When he/she says he/she is finished eating, I try to get my child to eat one more (two more, etc.) bites of food.** | Pressure |
| ***Excluded Items*** |  |
| 1. Do you let your child eat whatever s/he wants?* | Child Control |
| 2. At dinner, do you let this child choose the foods s/he wants from what is served?* | Child Control |
| 3. If this child does not like what is being served, do you make something else?* | Child Control |
| 4. Do you allow this child to eat snacks whenever s/he wants?* | Child Control |
| 5. Do you allow this child to leave the table when s/he is full, even if your family is not done eating?* | Child Control |
| 18. I withhold sweets/dessert from my child in response to bad behavior.** | Food as Reward |
| 49. I tell my child what to eat and what not to eat without explanation.** **R** | Teaching about Nutrition |

*Note.* Numbers correspond to the original items’ order in Musher-Eizenman & Holub’s paper. Items marked with a * utilize a 5-point response scale “never, rarely, sometimes, mostly, always”. Items marked with ** utilize a 5-point scale with different anchors, “disagree, slightly disagree, neutral, slightly agree, agree”. Items marked with an **R** were reverse coded.
